# Peptide-Based Nanogels for Pharmaceutical and Biotechnological Applications: From Fmoc-FF to Other Peptide Sequences

**DOI:** 10.3390/ph19040624

**Published:** 2026-04-15

**Authors:** Mariangela Rosa, Sabrina Marino, Giancarlo Morelli, Antonella Accardo, Carlo Diaferia

**Affiliations:** Department of Pharmacy, Centro Interuniversitario di Ricerca sui Peptidi Bioattivi “Carlo Pedone” (CIRPeB), University of Naples “Federico II”, Via Tommaso de Amicis 95, 80131 Naples, Italy; mariangela.rosa@unina.it (M.R.); sabrina.marino@unina.it (S.M.); gmorelli@unina.it (G.M.)

**Keywords:** nanogels, peptide nanogels, formulation, drug delivery, peptide materials, Fmoc-FF

## Abstract

Peptide-based materials represent a rapidly growing field in nanotechnology, bridging bottom-up self-assembly and top-down approaches for the development of functional nanostructures. Among these systems, peptide-based nanogels (NGs), namely nanogels in which peptides assume a structural role, have emerged as a promising class of injectable formulations. Typically characterized by a core–shell architecture, these systems are closely related to peptide hydrogels in terms of structural organization. This review provides a state-of-the-art overview of peptides used as core structural elements for NG formulation, focusing on the peptide building blocks employed, the main formulation methodologies, and their current applications, with particular emphasis on pharmaceutical ones. Their potential as drug delivery systems and stimuli-responsive platforms for controlled and targeted release is also reported. For clarity, the reported formulations are classified according to the chemical nature of the core-structuration peptide, distinguishing systems based on Fmoc-FF from those derived from other primary sequences, including Boc-protected tripeptides, dehydropeptides, and chemically crosslinked peptide assemblies.

## 1. Introduction

In the current landscape of nanotechnology tools for pharmaceutical applications, peptide-based materials represent a transformative area of research, driven by their inherent adaptability and the ability to bridge bottom-up and top-down fabrication methodologies. These materials have been proposed for drug delivery, vaccine development, tissue engineering, biosensors and gene therapy [1,2,3,4,5,6,7,8,9,10]. Researchers can produce a diverse array of nanodimensional architectures, including nanospheres, nanorods, nanotubes, nanostructured films, and nanodiamonds, tailored in their chemical composition, morphology, and functionality [11]. The formation of these architectures is strictly dictated by the peptide’s primary sequence, which requires balancing stability, structural organization and functional performance [12,13,14,15]. Factors such as charge, hydrophobicity, and secondary structure must be carefully considered, combined with the solvent environment, pH conditions and ionic strength [16]. All these parameters can significantly influence peptide interactions, the resulting structure and the drug delivery profiles.

**Figure 1:**
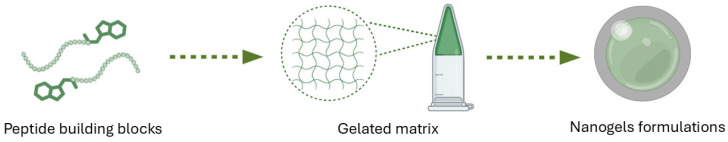
Schematic representation of peptide-based hydrogel and nanogel formation. Peptide building blocks, which can include aromatic or hydrophobic moieties, self-assemble in response to external triggers through non-covalent interactions, including hydrogen bonding, hydrophobic effects, and π–π stacking of aromatic moieties. The self-assembling processes lead to nanofiber formation. Their entanglement generates a three-dimensional hydrogel network. Nanogels are then obtained from this gel phase through appropriate formulation strategies, producing nanosized particles that retain the structural features of the parent matrix.

The multiscale and hierarchical arrangements of peptide architectures can also produce higher levels of organization, generating nanostructured bulk materials, including gels. Characterized by a three-dimensional network, gels are soft materials generated by trapped liquids, like water (hydrogels, HGs) or organic solvents (organogels, OGs), and exhibit viscoelastic no-Newtonian behavior [17,18]. The formation of peptide gels involves a series of progressive, well-defined steps. Initially, peptide gelator monomers are either dissolved or suspended in a suitable solvent. Upon introducing a gelation trigger (e.g., change in temperature, pH, solvent composition), self-assembly is initiated [19]. This process leads to the formation of long nanometer-scale fibers through non-covalent interactions. These nanofibrillar architectures subsequently entangle to create the gel space-spanning network at the micrometer scale, effectively trapping the solvent media [20,21,22]. The result is a self-supporting matrix that retains its structure even when inverted (Figure 1). The specific gelation trigger employed is crucial, as it influences not only the self-assembly process but also the final mechanical properties of the resulting gel. Different triggers can yield gels with varying characteristics, which can be tailored for specific applications [19]. Understanding these factors allows researchers to optimize gel formulations for desired outcomes in various fields, including materials science, biotechnology, and pharmaceuticals.

To expand the applications of gel matrices, injectable peptide-based nanogels (NGs) have emerged as a class of biomaterials related to HGs (Figure 1). The NG acronym is used for formulation in which peptides assume a structural rule as core building blocks. Architecturally, these systems often adopt a core–shell organization. The core typically consists of the dense, fibrillary aggregated peptide hydrogel network, while the hydrophilic shell provides colloidal stability and “stealth” properties to prevent opsonization. The methodology of formulation, involving strategies such as nanoprecipitation [23], inverse emulsion [24], or template-assisted polymerization [25], allows for the rigorous control of particle size (typically 20–200 nm) and mechanical properties. The hierarchical versatility of NGs gives them significant therapeutic advantages, including inherent biocompatibility, low immunogenicity, preservation of active pharmaceutical ingredient integrity, and the ability to degrade into non-toxic metabolites. Furthermore, their highly tunable chemistry permits the integration of stimuli-responsive motifs, which enable “on-demand” cargo release in response to the pathological microenvironment, such as the incorporation of pH-sensitive sequences for formulation swelling or disassembly in the acidic tumor microenvironment (pH 6.5–6.8) or endosomal compartments (pH 5.0–6.0). Similarly, the use of responsive peptide linkers may ensure drug release in specific biophases that overexpress particular enzymes, thereby maximizing therapeutic efficacy while minimizing off-target toxicity. By offering a sophisticated platform for controlled release and site-specific targeting through these tailored fabrication techniques, peptide-based nanogels may address critical challenges in oncology, gene therapy, and regenerative medicine, representing a transformative shift toward more precise and personalized medicine.

This review aims to provide an overview of the design principles, formulation routes and methodologies of peptide-based nanogels, highlighting their advantages as stimuli-responsive platforms for targeted and controlled drug delivery in modern pharmaceutical sciences. The classification into two main categories (Fmoc-FF-based and non-Fmoc-FF-related NGs) was made considering the chemical structure of the core-forming peptide building block. The distinction between Fmoc-FF-based NGs and other peptide-based ones is adopted here as a practical, literature-driven classification, since Fmoc-FF is the most extensively investigated peptide, associated with the largest number of reported nanogel formulations, preparation methods, and applications. The remaining systems highlight the broader structural diversity of other primary sequences explored for NG design.

## 2. Fmoc-FF Based Nanogel Formulations

Short and ultrashort peptides (2–6 residues) can self-assemble into supramolecular structures such as fibers, nanospheres and micelles, providing versatile platforms for controlled and efficient delivery of different active pharmaceutical ingredients (APIs) [26,27,28,29]. Supramolecular hydrogels can be formed from the self-assembly of one or more different low molecular weight gelators (LMWGs), differing in their chemical features. These matrices were used as bulk materials for NG formulations [30,31,32].

**Figure 2:**
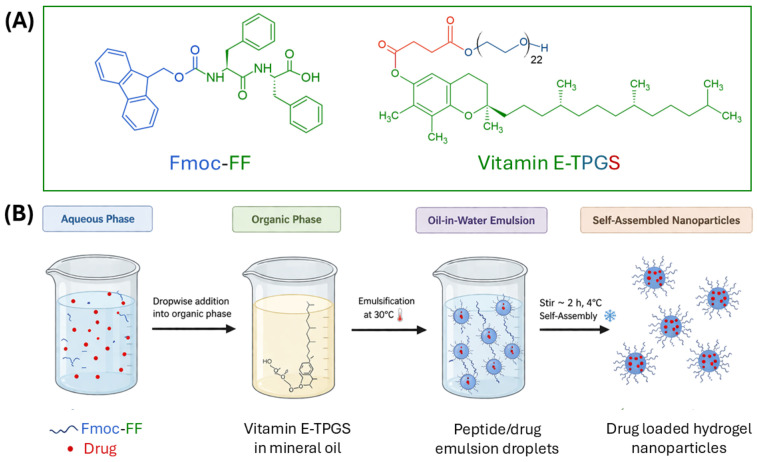
(**A**) Chemical structure of Fmoc-FF and Vitamin E-TPGS with name/structure color correlation. (**B**) Schematic representation of the inverse-emulsion method used to prepare Fmoc-FF hydrogel nanoparticles, namely nanogels. The aqueous peptide/drug phase was dispersed into the organic phase of mineral oil, generating emulsion droplets in which peptide self-assembly leads to final drug-loaded nanogels.

Fmoc-diphenylalanine (N^α^-fluorenylmethoxycarbonyl-diphenylalanine, Fmoc-FF, Figure 2A) is an LMWG, generating self-supporting matrices under mild conditions [33,34]. Chemically, Fmoc-FF is a diphenylalanine (FF) derivative, Fmoc (fluorenylmethoxycarbonyl) capped at the N^α^-terminus. Although Fmoc is generally used as an orthogonal protecting group in solid-phase peptide synthesis (SPPS), it was intentionally retained, playing a key functional role in gel organization and self-assembly. Gelation is driven primarily by a combination of π–π stacking between the fluorenyl groups, aromatic interactions between phenylalanine residues, and intermolecular hydrogen bonding along the peptide backbone. The supramolecular arrangement leads to the formation of supramolecular β-sheet-like nanofiber assemblies [35,36,37]. At a higher level of organization, mutual fiber entanglement generates the three-dimensional network able to immobilize large amounts of water at low critical gelation concentrations (CGC ~0.25 wt%). The Fmoc-FF gelation is highly sensitive to external parameters such as pH, solvent composition, temperature, and ionic strength, which modulate assembly kinetics and mechanical properties. Additionally, multicomponent gels can be generated by mixing Fmoc-FF with additional chemical or functional elements. This evidence is collected in phase diagrams for the gelation domain condition [19,20,21,22,35,38,39,40,41]. As a result, Fmoc-FF gels exhibit tunable stiffness, self-healing behavior, long shelf life, mechanical response and shapeability, and reversibility, making them widely used as model systems and functional scaffolds in biomaterials, pharmaceutical sciences and supramolecular chemistry research [42,43,44,45].

Fmoc-FF represents the most used peptide for NG formulations. Table 1 summarizes the main Fmoc-FF-based formulations, highlighting their preparation methods, structural features, loaded cargos, and principal pharmaceutical or biotechnological applications.

For the first time, in 2013, Ischakov et al. employed this aromatic dipeptide to develop a novel class of hydrogel nanoparticle (HNPs), formally nanogels, with the aim of designing nanocarriers for controlled drug delivery. Fmoc-FF-based nanoparticles were produced via an inverse water-in-oil (W/O) emulsion technique, in which the dipeptide, initially dissolved in an organic solvent (DMSO) and subsequently diluted in water at a concentration of 10 mg/mL (1.0 wt%), is dispersed into a mineral oil phase containing D-α-tocopheryl polyethylene glycol 1000 succinate (0.4% *wt*/*v*, vitamin E-TPGS, Figure 2A), as biocompatible surfactant [46]. E-TPGS, selected for its biocompatibility and non-immunogenic features, was expected to enhance nanoparticle stability, giving also the possibility of active targeted delivery due to surface functionalization via alcohol chemistry [47]. In order to promote the gelation process and the formation of the surrounding surfactant monolayer, the mixture underwent mechanical homogenization, where higher energy input enabled the dipeptide mixture to disperse and self-assemble into stable particle aggregates. NGs were then purified through centrifugation followed by multiple *n*-hexane washes. Finally, the collected NG fractions were resuspended in water or PBS before use (Figure 2B). Characterization by dynamic light scattering (DLS) revealed a bimodal size distribution (21.5 ± 1.3 and 225.9 ± 0.8 nm), which is attributed to the dynamic equilibrium between droplet fragmentation and droplet re-coalescence during emulsification. This evidence emphasized the influence of processing parameters (e.g., surfactant selection and homogenization energy) on the final nanoparticle size, and it was supported by the larger NGs size obtained using a lower energy input (via magnetic stirring). The NGs exhibit a negative average zeta (ζ) potential of approximately −25 ± 3 mV, ascribed to the presence of C-terminus carboxyl groups or to the coating effect of vitamin E-TPGS, contributing to the overall colloidal stability of the formulation. TEM analysis, revealing spherical structure of nanoparticles (average size under 100 nm), was also used to visualize encapsulated gold nanoparticles, thus demonstrating the hydrophilic nature of the nanoparticle core. The formulation also exhibited good stability upon lyophilization, both in the presence and absence of cryoprotectants (t-butyl alcohol), thanks to the E-TPGS, which also acts as an effective stabilizing agent. This evidence suggested that the Fmoc-FF NGs could be readily turned into a dry form, and resuspended prior to use, thus improving the shelf-life, storage and transportation conditions.

**Table 1:** Fmoc-FF-based nanogels details, indicating formulation strategy, characterization techniques, loaded active pharmaceutical ingredients (APIs), loading parameters, release data, and main applications. *Abbreviations*: nanogels (NGs); water-in-oil (W/O); D-α-tocopheryl polyethylene glycol 1000 succinate (vitamin E-TPGS); dynamic light scattering (DLS); zeta potential (ζ); transmission electron microscopy (TEM); doxorubicin (Dox); 5-fluorouracil (5-FU); circular dichroism (CD); nanoparticle tracking analysis (NTA); quantitative polymerase chain reaction (qPCR); fluorescein isothiocyanate (FITC); Fourier-transform infrared spectroscopy (FT-IR); dexamethasone (Dex); curcumin (Cur); scanning electron microscopy (SEM); reactive oxygen species (ROS); differential scanning calorimetry (DSC); cationic amphiphilic peptides (CAPs); encapsulation efficiency (EE); encapsulation rate (ER); small-angle X-ray scattering (SAXS). Data not present as not reported (NR).

System	Formulation	Characterization	API	Loading	Release	Efficacy/Application	Ref.
Fmoc-FF	Inverse W/O emulsion; vitamin E-TPGS.	DLS, ζ, TEM.	Dox, 5-FU	NR.	5-FU: ~50% (5 h) Dox: ~50% (20 h)	Drug-delivery; hydrophilic core demonstrated.	[46]
Fmoc-FF	Inverse emulsion, top-down, nanogelling-in-water; TWEEN60/SPAN60.	DLS, CS	Dox	NR.	~20% (>72 h); major release between 8 and 12 h.	Formulation optimization; drug delivery	[48]
Fmoc-FF	Top-down; TWEEN60/SPAN60	DLS, NTA, qPCR, confocal microscopy.	FITC	NR.	NR.	Selective uptake in MDA-MB-231 cells; caveolae-mediated internalization.	[49]
Fmoc-FF NGs	Top-down; TWEEN60/SPAN60.	FT-IR, CD, DLS, ζ, serum stability, hemotoxicity, cytotoxicity, SEM	Dex Cur	NR.	Cur: 5.7% (72 h) Dex: 60% in 72 h.	Selective uptake in leukemic cells; delivery and imaging	[50,51]
Fmoc-FF	Inverse W/O emulsion; TWEEN60/SPAN60	DLS, TEM, cytotoxicity, ROS assays, DSC	Naringin	NR.	NR.	Biocompatible in L929; cytotoxic to melanoma cells; topical antitumor potential.	[52]
Fmoc-FF/CAP	Top-down; TWEEN80/SPAN80	DLS, CD FT-IR, SAXS	AlexaFluor430	EE/ER depend on loading strategy.	~97% (adsorbed), ~26% (encapsulated) at 120 h.	Delivery of negatively charged molecules; potential nucleic-acid carriers.	[53,54]

Drug loading efficiency and release behavior of NGs loaded with doxorubicin (Dox) and 5-fluorouracil (5-FU) were evaluated under physiological conditions. The two drugs exhibited markedly different release profiles: 5-FU showed a rapid release, with approximately 50% released within 5 h and reaching a plateau shortly thereafter. On the contrary, Dox-loaded formulations revealed a slower and more sustained profile (drug release of 50% after 20 h). These differences were linked to the distinct drug physicochemical properties, specifically to the molecular weight and chemical structure. Stronger aromatic interactions and hydrogen bonding between the Dox anthracycline ring and the peptide matrix are believed to enhance its encapsulation and slow diffusion, while the lower molecular weight and weaker interactions of 5-FU lead to faster diffusion and a pronounced burst release [46].

**Figure 3:**
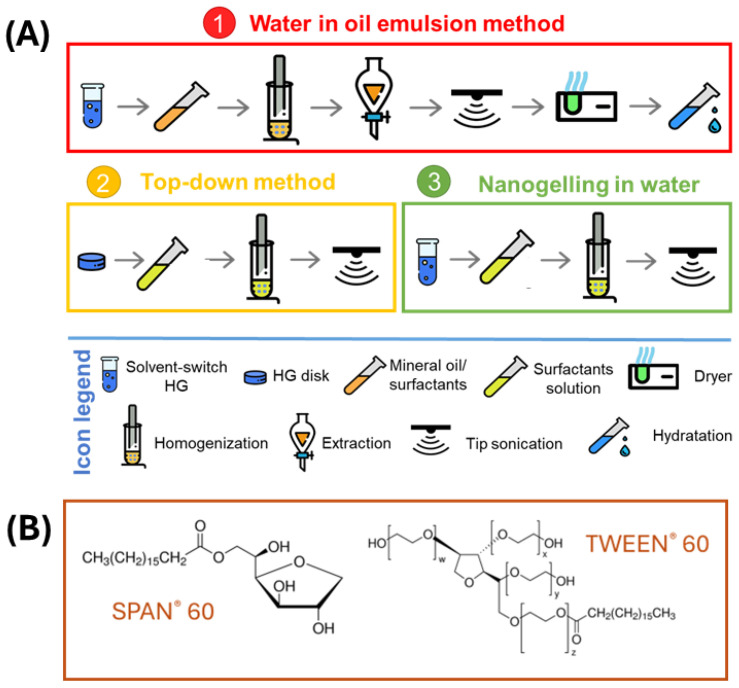
(**A**) Schematic icons representation of the three different strategies for Fmoc-FF nanogel formulation: (1) Water-in-oil emulsion methodology; (2) top-down methodology; and (3) “Nanogelling” in water. The icon legend is shown below. (**B**) Chemical structure of TWEEN60 and SPAN60 surfactants. Rearranged with permission from Ref. [48].

The same peptide building block, Fmoc-FF, was employed by Rosa et al. in 2020 for the preparation of stable NGs. [48] In this study, the authors investigated structural properties and stability of NGs obtained by three formulation routes: (i) water-in-oil emulsion technique (or reverse emulsion technique), (ii) top-down methodology, and (iii) nanogelling in water (Figure 3A). Specifically, the inverse-emulsion technique was reproduced as already reported [46]. The top-down methodology involves the formulation of nanogel particles from the macroscopic gel obtained in silicon molds. In this approach, nanogels are obtained starting from a preformed bulk hydrogel, which is subsequently subjected to mechanical homogenization and sonication in the presence of suitable surfactants, in order to reduce the macroscopic gel network into stable core–shell nanosized particles. Finally, nanogelling in water consists of mixing the metastable peptide/dimethyl sulfoxide (DMSO) sample in an aqueous medium of stabilizing agents before the gelling procedure is completed. First, the NGs were formulated reproducing the inverse-emulsion technique protocol previously described by Gazit’s group, showing a significant increase in size (from ≈250 nm to 1000 nm after 24 h). A formulative study was then conducted, preparing NGs while keeping constant the Fmoc-FF concentration (1.0 wt%) and varying the percentage of two biocompatible surfactants, TWEEN^®^60 and SPAN^®^60, using a water-in-oil emulsion technique (Figure 3B). The different molar ratios of these two surfactants allowed evaluation of the effect of the HLB values (4.7 < HLB < 14.9). Structural properties collected by DLS measurements highlighted a size-HLB direct correlation. Even if all the formulations show shelf stability at room temperature up to 30 days, the comparison among the intensity profiles of nanogels prepared according to the three methods identified HLB = 10 as the best value for obtaining a smaller, more monodisperse particle population (174 ± 82 nm). Among the three formulation methods, top-down was found to be the most reproducible one regarding feasibility, duration of procedure and high biocompatibility, avoiding organic solvent extraction and freeze-drying steps and the use of the ammonium sulphate procedure, generally used for Dox loading in liposomes.

**Figure 4:**
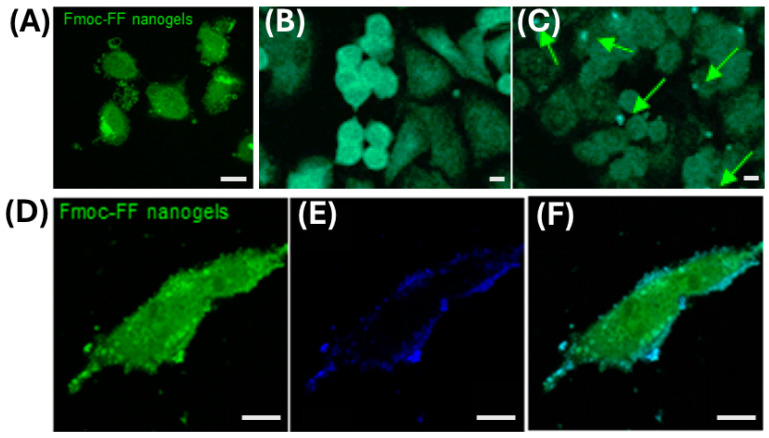
Intracellular localization confocal images of FITC-loaded Fmoc-FF NGs incubated with MDA-MB-231 cell line: (**A**) 1 h at 37 °C, (**B**) 1 h at 4 °C with PM staining of the FITC signal, and (**C**) switched to 37 °C for 10 min. Green arrows denoted the vesicular staining of the FITC signal. Colocalization evidence of NGs (**D**) and HSA-containing vesicles (**E**) and merged image (**F**) in MDA-MB-231 cells. Cyan reported the region of colocalization. Scale bars for all panels represent 14 μm. Adapted with permission from Ref. [49].

The Dox-releasing profile was evaluated in vitro using a dialysis method over 72 h, with 20% release. Most of the drug was released during 8–12 h (~50%), which is lower than values reported for Fmoc-FF nanogel prepared using E-TPGS surfactant by Gazit’s group (80% after 55 h). The same authors also evaluated the cytotoxicity effect of the Fmoc-FF NGs, prepared according to the top-down methodology, on a panel of breast cancer cells (SKBR3, MDA-MB-231, MDA-MB-361, and MDA-MB-453) or non-tumorigenic cell lines (MCF10-a and 3T3-L1), employed as control (see Figure 4) [49]. All cell subtypes were exposed to different peptide NG concentrations (5 × 10^−3^ wt% and 2.5 × 10^−3^ wt%) for a time span from 24 to 72 h. A cell-specific toxicity effect towards MDA-MB-231 was observed, with a maximum after 48 h for both concentrations. Surprisingly, a reversible growth arrest was observed at lower peptide concentration after 48 h of treatment with a recovery after 72 h reaching a viability comparable with the untreated controls. This effect was also obtained by employing a diluted concentration (6.25 × 10^−4^ wt%). The flow cytometry (FCM) confirmed the reversible growth-arrest effect on MDA-MB-231 during the first 24 h of incubation, supported by the increasing levels of cyclins E and B with an arrest in the S phase of the cell cycle. By starting from the awareness of the endocytic machineries involved for Fmoc-FF nanogels cellular uptake, such as endocytosis, phagocytosis and pinocytosis, but also perforation, due to a possible direct contact toxicity on plasma membrane (PM), the authors chose to follow the entry pathway with FITC-loaded NGs. Collectively, all the in vitro data allowed us to conclude that the uptake of Fmoc-FF NGs is mediated by caveolae. The Fmoc-FF nanogels’ selectivity towards MDA-MB-231 was further confirmed by qPCR of caveolin transcript, which highlighted high expression levels of caveolin-1 with respect to other tested cell subtypes [49].

The same formulation route was successively adopted to produce Fmoc-FF HGs and NGs encapsulating poorly soluble drugs like dexamethasone (Dex) and curcumin (Cur) [50,51]. In both studies, hydrogels (1.0 wt%) were formulated according to the DMSO/water solvent-switch method, including hydrophobic drug from a stock solution (200 mg/mL, final concentration in gel = 10 mg/mL). This strategy allowed to encapsulate an amount of drug significantly higher than its water solubility, without the observation of syneresis phenomena. However, it can be observed that there are different gelation kinetics for the encapsulation of Dex and Cur into the hydrogelated matrix, with a significant increase in the gelation time only for Cur with respect to the empty Fmoc-FF HG (1 h *versus* 2 min). On the other hand, the inclusion of both drugs causes an increase in the gel’s mechanical rigidity (G′ = 67.9 and 11.8 kPa, for Dex and Cur, respectively). This increase in stiffness can be attributed to the tight hydrophobic matrix/drug interaction pathway.

The corresponding injectable NGs, formulated according to top-down methodology, showed reliable stability after 90 days, in terms of size and ζ potential (~200 nm and ~−30 mV, respectively). FT-IR, Thioflavin T (ThT) assays and circular dichroism (CD) spectra measurements confirmed that drug encapsulation on both HGs and NGs does not alter the structural organization. Interestingly, the release profiles observed for the two drugs from the NG, having the same composition and structure, are very different, with a release of 5.7% for Cur and 60% for Dex over 72 h. This different behavior can be explained by assuming that the two drugs have different non-covalent interactions within the inner core of the NG. Before carrying out in vitro cellular assays, the stability and hemotoxicity of the NGs formulation were demonstrated by incubating FITC-filled NGs with human serum (72 h at 37 °C) at three concentrations (1.3 × 10^11^, 2.6 × 10^10^ and 1.3 × 10^10^ particles/mL). Subsequently, NGs loaded with Dex or Cur were tested on the leukemic B-cells model RS4, 11 or thyroid cancer (K1 and CAL62) cell lines, which overexpress caveolin-1 receptors. In vitro cytotoxicity assays confirmed that toxic effects were only related to the drug. Furthermore, by analyzing the cell cycle, it was found that the growth of Dex-treated cells was arrested by reducing G1 and increasing G2-M phases. Cytofluorimetric analysis, after 120 min incubation of FITC-filled NG with both RS4; 11 and healthy cells, showed the rapid and selective internalization in leukemic cells, thus suggesting the possible use of Fmoc-FF NG for passive targeting in onco-hematological diseases.

The same Fmoc-FF NG formulation, loaded with the hydrophobic phytochemical naringin (NAR), was also proposed by Secerli et al. as potential nanodrugs for the treatment of melanoma. NAR is a natural compound from citrus with anticancer and anti-inflammatory properties, but poor bioavailability. Structural characterization through DLS and transmission electron microscopy (TEM) revealed that NGs have a nearly spherical organization with dimensions in the 200–300 nm range. Regarding drug delivery performance, the nanogels demonstrated high biocompatibility in healthy mouse fibroblast (L929) cells while showing potent cytotoxic effects against human melanoma line SK-MEL-30 (calculated IC50 of ~69 µg/mL). Furthermore, treatment with these nanogels led to a concentration-dependent increase in reactive oxygen species (ROS) production in melanoma cells, which, coupled with an increase in tyrosinase enzyme activity and a decrease in inflammatory biomarkers (NF-κB, TNF-α, and IL-8), highlighted their potential as an effective topical therapy for melanoma [52].

**Figure 5:**
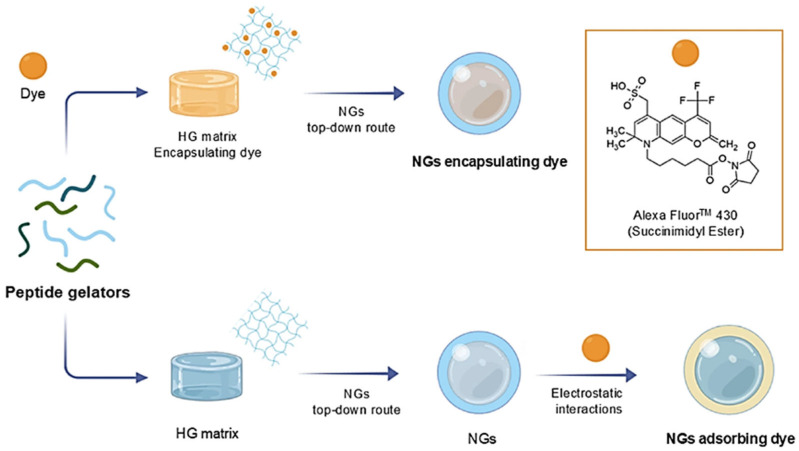
Strategies’ schematic representation of encapsulation or adsorption for AlexaFluor430. Reproduced with permission from Ref. [54].

Fmoc-FF dipeptide was also used in combination with additional components to formulate mixed NGs with enhanced properties. For example, in 2025, Rosa et al. used Fmoc-FF in combination with cationic amphiphilic peptides (CAPs) in multicomponent NGs for the delivery of negatively charged APIs like nucleic acids [53]. CAPs, with a general formula Cn-(GK)3, contained the hexapeptide (GK)3, which alternates three Gly and three Lys residues and an alkyl chain with a number of carbon atoms (Cn) ranging from 8 to 18 carbon atoms. NGs, coated by TWEEN^®^80/SPAN^®^80 (HLB = 10), were prepared according to the protocol previously described [48]. DLS characterization highlighted that NGs containing capric (C10-), lauric (C12-) and myristic C14-(GK)3 do not have size characteristics compatible with injection through a needle (polydispersity index > 0.350). Instead, Fmoc-FF/C16-(GK)3 and Fmoc-FF/C18-(GK)3 NGs showed a monomodal DLS profile (with a main diameter of 139 and 102 nm, respectively) and a more homogeneous aggregate population (PDI = 0.202 and 0.243, respectively). In addition, due to the positive ζ potential values (+38 and +51 mV for C16- and C18-containing formulations, respectively), these NGs were found to be stable over time (up to 30 days). Moreover, preliminary in vitro assays performed on HEK-293 cells confirmed that these NGs were not toxic until an incubation time of 72 h. To evaluate the drug delivery ability of these NGs, they were decorated with increasing amounts of AlexaFluor430 dye (succinimidyl ester) with two different strategies: encapsulation into the inner core and surface absorption via electrostatic interactions (Figure 5) [54]. According to DLS measurements, all the formulations, except those with a higher amount of adsorbed dye, were found to be stable with a lower PDI associated with samples prepared with the adsorption method. Results pointed out that the ER% (encapsulation rate) and EE% (encapsulation efficiency) values for both C16 and C18 NG formulations strongly depend on the loading methodology (encapsulation versus adsorption). As expected, the amount of released dye, evaluated by fluorescence spectroscopy, was found to be dependent on the loading method, with a percentage of ~97 and ~26% for adsorbed and encapsulated formulations, respectively, after 120 h.

The slow release of the encapsulated dye can be explained by a delayed water exchange between the internal core and the external solution.

**Figure 6:**
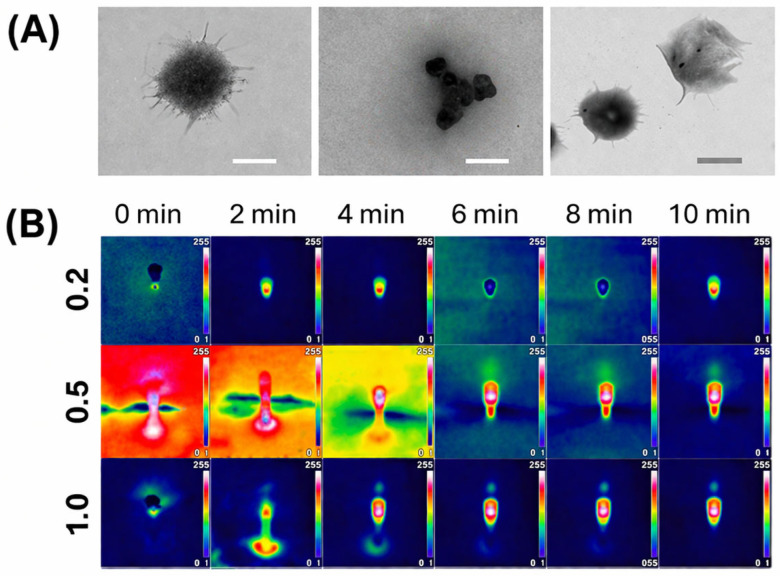
(**A**) TEM characterization of formulations, Fmoc-FF nanogel (on the left), AuNS-embedded NGs (middle), and AuNP-embedded Fmoc-FF ones (on the right). Scale bars correspond to 200 nm. (**B**) Thermal camera images of the AuNS-embedded Fmoc-FF nanogel under laser illumination for different times and powers of 0.2, 0.5 and 1.0 W/cm^2^. Rearranged with permission from Ref. [55].

In this context, another example of Fmoc-FF-based NGs was also reported by Erdogan et al. These NGs, prepared via a simple dispersion approach, were loaded with gold nanoparticles (AuNPs), gold nanostars (AuNSs) and/or Epirubicin (EPI). In detail, the formulations were obtained by inducing aqueous gelation of the dipeptide followed by incorporation of pre-synthesized gold nanoparticles. The latter were found homogeneously embedded within the nanofibrous network without disrupting gel formation. Characterization performed using FTIR, UV–vis spectroscopy, and X-ray diffraction confirmed both the peptide self-assembly and the presence of stable AuNPs, while electron microscopy (TEM, Figure 6A) revealed a well-defined nanofibrous structure. The authors demonstrated the capability of AuNS-embedded and AuNP-decorated Fmoc-FF NGs to allow a sustained release profile for EPI and a marked increase in release when NGs are exposed to light irradiation (808 nm NIR—near-infrared radiation) due to the photothermal effect of the plasmonic gold nanoparticles (Figure 6B). This evidence suggested a possible ON/OFF drug release mechanism. Mechanistically, the ON/OFF release profile can be ascribed to the photothermal conversion of the plasmonic gold nanoparticles under NIR irradiation. The 808 nm radiation can generate local heating, transiently increase the matrix permeability, thus promoting EPI diffusion. Then, once the irradiation is switched off, the thermal stimulus terminates, and the release rate decreases consequently.

Human neuroblastoma (SH-SY5Y) and mouse fibroblast (L929) cell lines were used for biocompatibility studies. In addition, the genotoxicity profile of the NGs was also investigated in SH-SY5Y cells [55].

## 3. Non-Fmoc-FF Related Nanogels

Beyond Fmoc-FF, a series of different short and ultrashort peptides capable of gelation were explored as building blocks for the preparation of NGs for different applications (see Table 2)Even in these peptides, often one of the termini bears a protecting group like Fmoc-, Ac- (Acetyl), Boc- (tert-butyloxycarbonyl) and Nap- (Naphthyl-), typically used in the solid-phase peptide synthesis. Among them, one of the first examples reported in 2015 describes the formulation of an NG based on the ultrashort capped peptide Boc-Pro-Phe-Gly-OMe [56]. NGs were prepared using the inverse water-in-oil emulsion technique in the presence of vitamin E-TPGS and loaded with several hydrophobic model drugs (aspirin, eosin and curcumin). The encapsulation of the drugs was achieved by co-dissolving each drug with the peptide in methanol, followed by the addition of water, overnight stabilization and lyophilization, driven by hydrophobic interactions between the core and the API. As expected, drug encapsulation caused an increase in the NGs’ size from 119.6 nm to 135.5 nm, 217.1 and 143.9 for eosin, aspirin, and curcumin-loaded NGs, respectively. A sustained release was observed for all the drugs and attributed to diffusion-driven transport from the hydrophobic core of the NG. Moreover, a non-specific toxicity towards MCF-7 human breast cancer and HaCaT human keratinocyte was observed for all the vitamin E TPGS stabilized nanostructures. These data were attributed to an interaction of the non-ionic surfactant with the cell membrane, resulting in a cell surface morphology alteration.

Successively, the same authors synthesized a new NG formulation, based on the peptide Boc–Pro–ΔPhe–Gly–Ome, in which the phenylalanine (Phe) residue is replaced with the unnatural amino acid Δ-dehydrophenylalanine (ΔPhe) to enhance the in vivo stability of the nanostructure [57]. Indeed, it is well-known from the literature that this uncoded amino acid can induce conformational constraints and improve resistance to enzymatic degradation. Additionally, ΔPhe can act as a β-sheet-breaking residue, thereby inhibiting amyloid formation [58,59].

The resulting NGs were loaded with hydrophobic molecules like curcumin and ornidazole and externally stabilized using vitamin E–TPGS.

**Figure 7:**
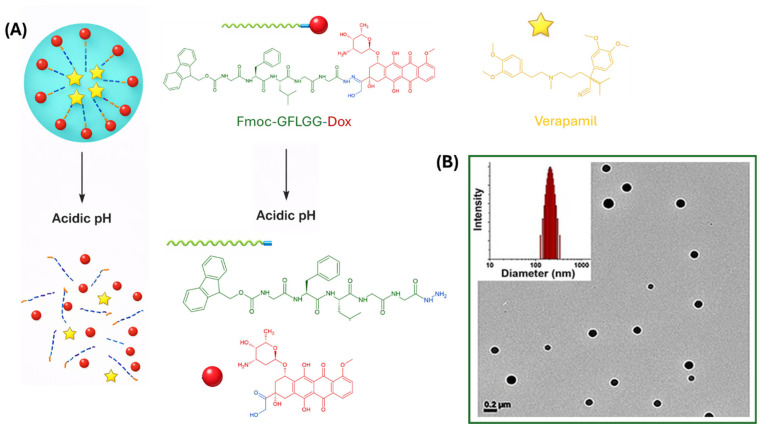
(**A**) Chemical structure of Fmoc-GFLGG-Dox and verapamil. Schematic aggregation/release mechanism. (**B**) NGs TEM microphoto and size diagram histogram. Arranged with permission from Ref [60].

Another ultrashort peptide, Fmoc-GFLGG, capped at the N-terminus with the fluorenyl protecting group, was used by Lyu and co-workers to promote the formation of acid-responsive NGs [60]. In this case, the NG is obtained in combination with verapamil (VER), a potent inhibitor of the ATP-dependent efflux transporter P-glycoprotein (P-gp), whose overexpression plays a key role in reducing intracellular drug accumulation and promoting multidrug resistance (MDR). NG fabrication involved the conjugation of Dox to the Fmoc-GFLGG peptide sequence via an acid-labile hydrazone linker, yielding a peptide–Dox (PD) prodrug (Figure 7A). The conjugate self-assembles in aqueous solution (pH 7.4) upon heating, followed by cooling. DLS and TEM analyses revealed spherical NGs with an average hydrodynamic diameter of approximately 199 nm, while the presence of VER did not alter the self-assembly behavior (Figure 7B). Fluorescence studies carried out on the NGs as a function of pH allowed to demonstrate the NG disassembly under acidic conditions (pH 5.0), with 70.4% of VER released within 10 min and sustained Dox release reaching 61.7% after 96 h, compared with only 17.1% Dox release at pH 7.4. Cellular uptake studies in non-resistant A2780 and Dox-resistant A2780/ADR ovarian cancer cells showed effective internalization of PD/VER NGs in both cell lines, with initial lysosomal localization followed by nuclear accumulation. In contrast, free Dox fluorescence was detected only in non-resistant cells. Calcein AM assays further confirmed P-gp inhibition and enhanced intracellular drug accumulation mediated by the NGs. Cytotoxicity assays demonstrated a dose-dependent antitumoral effect of PD/VER nanogels in both sensitive and resistant cells, while peptide and VER alone were non-toxic. Notably, in A2780/ADR cells, PD/VER nanogels exhibited significantly enhanced efficacy compared with free Dox, resulting in a 6.8-fold increase in anti-MDR activity.

Similarly, Zhang et al. proposed an NG based on the self-assembly of an ultrashort peptide derivatized with the Fmoc group (Fmoc-Tyr(H_2_PO_3_)-OH) and loaded with a magnetic enzyme to mimic the neutrophil-based innate immune response for anticancer therapy [61]. The system combines low-intensity magnetocaloric activation to elevate intracellular ROS in cancer cells with sustained chloroperoxidase (CPO) enzymatic oxidation, enabling a programmed magnetocaloric–enzymatic tandem therapy (METT). The increase in intracellular ROS in cancer cells is achieved through the self-assembly of the peptidic building block with magnetic nanoparticles (MNPs) that were found to elevate the H_2_O_2_ levels in cancer cells under programmed alternating magnetic field (AMF) [62].

Subsequently, CPO was loaded to catalyze the conversion of H_2_O_2_ into singlet oxygen (^1^O_2_), enhancing the therapeutic effect [63]. These MNP-CPO@NGs were obtained through a surface acid phosphatase (AP)-triggered self-assembly of Fmoc-Tyr(H_2_PO_3_)-OH molecules around the MNP core, followed by non-covalent immobilization of CPO. TEM and DLS analyses showed that the MNPs had diameters of ~85 nm (TEM) and 120 ± 14.5 nm (DLS), while MNP-CPO@Nanogels showed an increase in size (~100 nm and 187 ± 18.3 nm, for TEM and DLS, respectively). Encapsulation of CPO induced a marked shift in ζ potential, from a positive value (+39.2 ± 6 mV) to a negative one (−24.5 ± 3.9 mV). The therapeutic mechanism of METT was systematically validated through in vitro cellular studies in U251 glioma cells, revealing a pronounced synergistic effect between magnetic hyperthermia and enzymatic therapy, which was evident in both cancer cell destruction in vitro and tumor regression in vivo.

In 2021, Xia et al. reported another example of self-assembled SiO_2_@MCSGs as an efficient multienzyme-mimicking nanoplatform for theranostic applications (bioresponsive tandem catalysis and PA tumor imaging) [64]. The authors used a bioinspired strategy to construct metal-coordinated NGs (MCSGs) that mimic natural matrix-associated multienzyme complexes. The system was built by the in situ self-assembly of di-lysine-coordinated iron (Fe(Lys)_2_)-functionalized peptide gelator, NapFFE, on the surface of SiO_2_ nanoparticles, chosen for their chemical stability, biocompatibility, ease of functionalization, and lack of intrinsic catalytic interference. The formation of SiO_2_@MCSGs was achieved through an amidation-induced protonation process, in which carboxyl-modified silica reacted with amino groups of NapFFE-Fe(Lys)_2_, leading to peptide deposition on the nanoparticle surface. Subsequent π–π stacking interactions between naphthalene and phenylalanine residues promoted in situ hydrogelation. Morphological and physicochemical characterization confirmed the formation of a core–shell structure with an ~10 nm nanogel layer, good colloidal stability in physiological media, successful grafting and efficient ABTS loading. The monoatomic and highly dispersed Fe(Lys)_2_ active centers created a nanocompartmental structure analogous to natural extracellular-matrix-associated multienzyme assemblies, resulting in enhanced catalytic efficiency. SiO_2_@MCSGs exhibited dual superoxide dismutase (SOD)-like and peroxidase (POD)-like activities. After loading ABTS, the system could responsively convert O_2_^−^ into H_2_O_2_ via SOD-like activity and subsequently catalyze ABTS oxidation through POD-like activity, generating a product with strong near-infrared (NIR) absorption suitable for photoacoustic (PA) imaging. Catalytic studies demonstrated superior SOD- and POD-like activities compared to free Fe(Lys)_2_, supported by EPR evidence of Fe(III) intermediates and Michaelis–Menten kinetics. The catalytic efficiency increased under acidic conditions, consistent with the tumor microenvironment. In vitro PA imaging showed a concentration-dependent increase in NIR absorption and PA signal with increasing H_2_O_2_ levels (5–50 µM). The in vivo experiments revealed efficient cellular uptake, negligible cytotoxicity up to 200 µg mL^−1^, and strong PA signals localized in tumor tissue, peaking at 2 h post-injection. The circulation half-life was 1.48 h, and histological analysis confirmed good biocompatibility with no observable organ damage.

**Figure 8:**
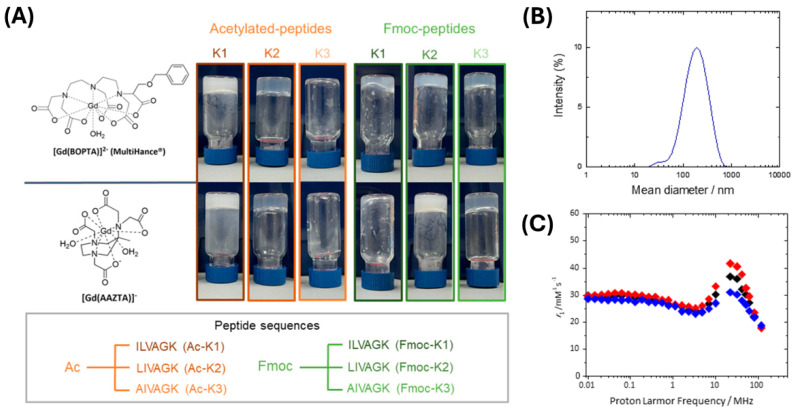
(**A**) Peptide one code letter primary sequences and Gd(III) complexes ([Gd(BOPTA)]^2−^ and [Gd(AAZTA)]^−^) used for hydrogel formulations (reported as inverted test tube samples). (**B**) [Gd(BOPTA)]^2−^ loaded Fmoc-K2 NG DLS profile. (**C**) NGs formulation ^1^H NMRD profile at different temperatures: 283 (blue), 298 (black) and 310 K (red). Rearranged with permission from Ref. [65].

With the aim of developing novel supramolecular contrast agents for diagnostic applications in magnetic resonance imaging (MRI), Rosa et al. formulated cationic peptide NGs that, by taking advantage of non-covalent electrostatic interactions, allowed encapsulation of negatively charged Gd(III) complexes (Figure 8A). Following the previously described top-down methodology [48], the authors encapsulated linear ([Gd(BOPTA)]^2−^) and macrocyclic ([Gd(AAZTA)]^−^) [65] Gd(III)-complexes in different hydrogels obtained by the self-assembly of N^α^ Fmoc- or acetyl (Ac) protected cationic peptides (K1, K2 and K3). Among all the matrices, Fmoc-K2+[Gd(BOPTA)]^2−^ showed a higher relaxivity (up to five-fold) compared with the free Gd-complex. Moreover, the increase in the relaxivity value as a function of the temperature suggested a limited water exchange due to a reduced diffusion of water within the gel matrix. Based on its favorable relaxometric profile, the corresponding Fmoc-K2+[Gd(BOPTA)]^2−^ nanogel was prepared by employing TWEEN85/SPAN85 in 89/11 (*w*/*w*) (HLB = 10). The resulting NGs exhibited a mean diameter of ~190 nm (Figure 8B), compatible with systemic administration. Moreover, CD spectra indicated the preservation of the β-sheet organization and the presence of strongly twisted β-sheets. Furthermore, the nanogel exhibited an even higher relaxivity (r_1_ = 36.8 mM^−1^ s^−1^ at 20 MHz and 298 K) compared to the corresponding hydrogel, attributed to reduced rotational freedom of the encapsulated Gd complexes within the nanostructured matrix (Figure 8C).

**Figure 9:**
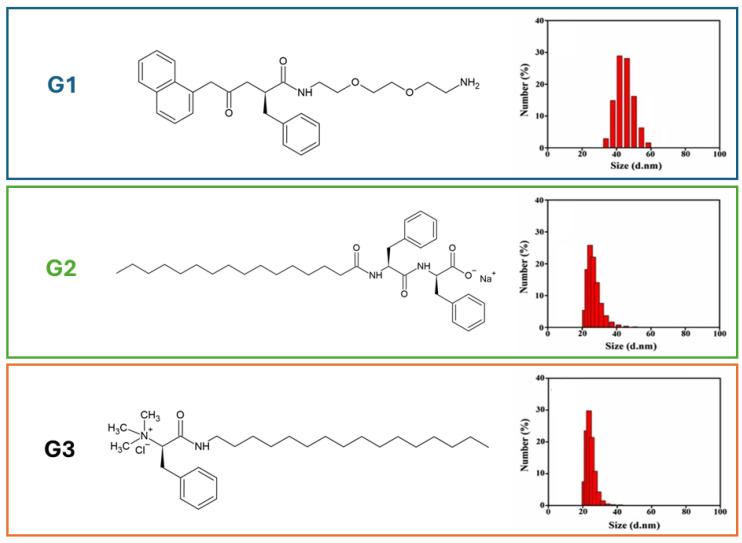
Chemical structures of G1, G2 and G3. On the right, DLS plot of nanogels prepared at HLB = 10.

Differentially charged peptide-based NGs neutral (NG1), negative (NG2), and positive (NG3) were also successively proposed by Pal et al. [66] to act as aqueous platforms for lipase, which is a ubiquitous enzyme that catalyzes fat hydrolysis to fatty acids and glycerol [67]. Water-insoluble substrates, such as lipids, are typically hydrolysed by lipases at the water–lipid interface, but their access to the enzyme active site in aqueous media is limited. Although various micro-heterogeneous systems have been developed to enhance lipase activity, these environments often compromise the enzyme’s native structure due to exposure to non-aqueous conditions [68]. In this context, NGs, owing to their hydrophilic and biocompatible nature, are expected to provide a favorable microenvironment that preserves the native conformation of the enzyme, while their high surface area may further enhance catalytic performance by facilitating substrate–enzyme interactions. NG1, NG2 and NG3 were formulated using three different amphiphilic hydrogelators, namely G1, G2 and G3 (Figure 9), employing the “nanogelling-in-water” approach [48] involving dispersion of the corresponding hydrogels in aqueous solutions of SPAN60 and TWEEN60, followed by bath sonication and ultrasonic homogenization.

DLS analyses revealed that hydrodynamic diameters of NGs decreased from 65 nm to 10 nm as the HLB (hydrophilic–lipophilic balance) increased from 4.7 to 14.9. Moreover, enhanced surfactant–gelator interactions at higher hydrophilicity led to the stabilization of smaller nanogels. Different microscopic techniques (SEM, TEM, AFM) were used to confirm the formation of NGs at an intermediate HLB value of 10, and all were consistent with the DLS data. Depending on the HLB, even the ζ values drastically changed (from −4.61 to 1.78) for NG1, (from −30.5 to −12.8) for NG2, and from (8.09 to 28.7) for NG3. These NGs, and the negatively charged NG2, were found to significantly enhance the catalytic activity of *Chromobacterium viscosum* (CV) lipase activity compared to surfactant mixtures, aqueous buffer, and CTAB (cetyltrimethylammonium bromide) micelles. Successively, in 2024, the same authors developed a novel nanoconjugate platform (NG-FACD) by integrating G3-based NGs with fluorescent carbon dots (CDs) acting as diagnostic probes. This hybrid system was designed as a theranostic strategy to overcome cancer drug resistance by combining multiple therapeutic modalities. NG-FACD is formed through electrostatic interactions between the positively charged NG3 and the negatively charged folic acid-functionalized blue-emitting carbon dots (FACD). The presence of folic acid confers target specificity to the NG toward folate receptor–overexpressing (FR+) cancer cells. Based on the synergistic potential of photodynamic therapy (PDT) and chemotherapy, NG-FACD was engineered for the co-delivery of riboflavin (RbF), a photosensitizer capable of reactive oxygen species (ROS) generation, and paclitaxel (PTX), a microtubule-stabilizing chemotherapeutic agent. The system showed selective uptake and cytotoxicity toward FR+ melanoma B16F10 cells compared to FR−A549 and non-tumoral HEK293 cells. NG-FACD exhibited significantly enhanced loading efficiency for both RbF and PTX compared to individual nanocarriers, resulting in enhanced cytotoxicity (~1.9–2.8-fold) toward FR+ cells, as well as superior efficacy compared to free drugs and single drug-loaded systems. The conjugated architecture also enabled higher ROS generation under visible-light, consistent with the increased RbF loading. Moreover, biocompatibility studies demonstrated acceptable cell viability, while competitive inhibition with free folic acid confirmed receptor-mediated cellular uptake. Finally, nuclear morphology analysis revealed pronounced apoptotic features in B16F10 cells treated with dual drug-loaded NG-FACD, confirming the enhanced anticancer efficacy achieved through the synergistic combination of PDT and chemotherapy [69].

**Figure 10:**
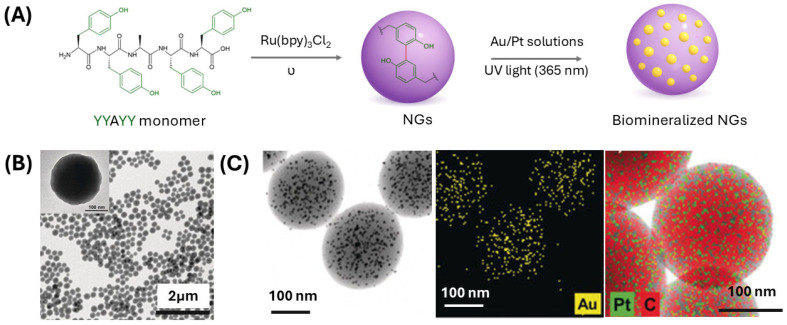
(**A**) Sequential representation for Tyr-based cross-linked NGs from tyrosine-rich peptide monomer (YYAYY), and its biomineralization via permeability uptake. (**B**) TEM micrograph of NG (1.0 mg/mL) and HR-TEM surface (insert). (**C**) From the left: STEM images of biomineralized Au–peptide hybrids, mapping of Au and Pt/C visualization. Rearranged with permission from Ref. [73].

With the aim of stabilizing NGs, a covalent self-assembly-based approach [70,71,72] was proposed by Min et al. [73]. The peptide NG is prepared using a tyrosine-rich short peptide monomer (YYAYY, Figure 10A). The covalent NG was generated by a one-step visible-light (6 min of exposure) mediated by a Ruthenium complex ([Ru-byp_3_]Cl_2_). The photo-crosslinking of the tyrosine-rich peptide monomer leads to dityrosine-based covalent assembly. The covalent NG, having an average diameter of 235 ± 31 nm, exhibited good stability across different pH (4–6) and solvent conditions (including acetonitrile, toluene, dichloromethane, and tetrahydrofuran). In addition, it was observed that the NG average size can be tuned as a function of the peptide monomer concentration, rising from 131 to 315 nm by moving from 0.2 to 2 mg/mL (Figure 10B) Due to the redox-active features, the resulting formulation can also act as a biocompatible artificial nanoreactor, enabling the in situ formation of uniformly dispersed metal nanoparticles of gold or platinum from AuCl_3_ and H_2_PtCl_6_ solutions (0.25 mM), respectively. The biomineralization process was performed via UV light exposure at 365 nm for 15 min. The final metal–peptide hybrid nanogels exhibited high water dispersibility, structural stability, uniform nanoparticle size and catalytic activity (Figure 10C).

Analogously, Kimura et al. also developed a radiation-crosslinked peptide NG for pancreatic cancer diagnosis [74]. Radiation-based modification of soluble proteins and peptides has been widely employed to produce functional biodevices ranging from the nano- to the macro-scale, without the need for cytotoxic chemical agents [75]. In contrast to solid-state proteins or peptides, which predominantly undergo degradation due to the direct action of ionizing radiation, these direct effects are largely mitigated in aqueous solutions because water absorbs most of the radiation energy. In aqueous media, both direct and indirect effects of ionizing radiation may contribute to peptide/protein modification. Nevertheless, because water can absorb a significant fraction of the radiation energy, the subsequent radiolysis products (e.g., hydroxyl radicals under aerated conditions) are often related to the observed structural changes [76,77].

**Figure 11:**
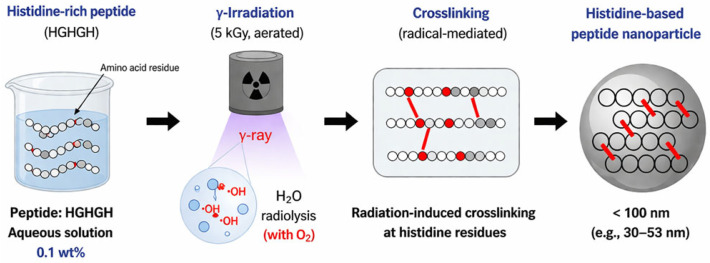
Schematic representation of γ-radiation-assisted formulation for His-based peptide nanogels. HGHGH-rich sequences (0.1 wt%) are exposed to γ-irradiation (5 kGy, aerated conditions), leading to water radiolysis. The generated reactive species (•OH) promote His radical formation, resulting in an intermolecular crosslinking and formation of nanosized peptide nanogels.

Beyond tyrosine and phenylalanine residues, histidine residues have also been found to play a key role in radiation crosslinking [78]. Accordingly, histidine-rich peptide sequences (HGGGHGGGH (H9), HGGGGGHGGGGGH (H13), HGHGH (H5) were rationally designed and synthesized to generate γ-rays radiation-crosslinked NGs with sizes below 100 nm and a negative surface potential (Figure 11). These two features are critical for promoting efficient accumulation of the system in pancreatic cancer cells [79,80]. In these systems, aqueous solutions (0.1 wt%) of the histidine-rich peptides H5, H9, and H13 were irradiated with γ-rays under aerated conditions (dose of 5 kiloGray from a ^66^Co source), yielding nanogels with average diameters of 53, 49, and 30 nm, respectively. Mechanistically, crosslinking is mainly driven by hydroxyl radicals generated by water radiolysis, which react with His imidazole groups, producing peptide radicals that subsequently dimerize into a three-dimensional nanogel network. The NG ζ was shifted from positive to negative through fluorescent labeling, resulting in a surface potential of −25.0 mV for the fluorescently labeled H9 nanogels. Notably, this surface modification enabled efficient accumulation of the nanogels in pancreatic cancer cells.

**Table 2:** Overview of peptide-based nanogels not based on Fmoc-FF reported in the literature. The table summarizes the main non-Fmoc-FF peptide nanogel formulations, including formulation strategy, characterization methods, loaded active pharmaceutical ingredients or functional cargos, loading and release features, and main pharmaceutical or biotechnological applications. Abbreviations: NGs, nanogels; W/O, water-in-oil; vitamin E-TPGS, D-alpha-tocopheryl polyethylene glycol 1000 succinate; DLS, dynamic light scattering; TEM, transmission electron microscopy; Dox, doxorubicin; VER, verapamil; AP, acid phosphatase; MNP, magnetic nanoparticles; CPO, chloroperoxidase; ROS, reactive oxygen species; SiO_2_, silica; MCSGs, metal-coordinated supramolecular gels; EPR, electron paramagnetic resonance; Gd(III), gadolinium(III); CD, circular dichroism; NMRD, nuclear magnetic relaxation dispersion; SEM, scanning electron microscopy; AFM, atomic force microscopy; PA, photoacoustic; NR, not reported.

System	Formulation	Characterization	API	Loading	Release	Application	Ref.
Boc-Pro-Phe-Gly-OMe	Inverse W/O emulsion; vitamin E-TPGS	DLS	Aspirin, eosin, Cur	NR	Sustained release	Drug delivery; hydrophobic cargo encapsulation	[56]
Boc-Pro-ΔPhe-Gly-OMe	Inverse W/O emulsion; vitamin E-TPGS	DLS	Cur, ornidazole	NR	Sustained release	Drug delivery; improved stability through dehydrophenylalanine incorporation	[57]
Fmoc-GFLGG/Dox	Self-assembly in water by heating/cooling; co-loaded with VER	DLS, TEM, fluorescence studies	Dox, VER	NR	VER: 70.4% in 10 min at pH 5.0; Dox: 61.7% in 96 h at pH 5.0; 17.1% at pH 7.4	Acid-responsive intracellular drug release; overcoming multidrug resistance	[60]
Fmoc-Tyr(H_2_PO_3_)-OH	AP-triggered self-assembly around MNP core; non-covalent CPO immobilization	DLS, TEM, ζ	CPO on MNP core	NR	NR	Magnetocaloric–enzymatic tandem therapy; ROS-mediated anticancer activity	[61]
NapFFE-Fe(Lys)_2_ NGs/SiO_2_@MCSGs	In situ self-assembly on SiO_2_ surface through amidation-induced protonation and hydrogelation	DLS, TEM, catalytic studies, EPR	ABTS, Fe(Lys)2 catalytic centers	NR	Responsive catalytic activation in tumor-like environment	Multienzyme-mimicking theranostic platform; tandem catalysis and PA imaging	[64]
Fmoc-/Ac-protected cationic peptide	Top-down methodology; TWEEN80/SPAN80	DLS, CD, relaxometric analysis, NMRD	[Gd(BOPTA)]^2−^, [Gd(AAZTA)]^−^	Electrostatic encapsulation	NR	Magnetic resonance imaging contrast enhancement	[65]
Differently charged peptide amphiphile NGs (NG1, NG2, NG3)	Nanogelling-in-water; SPAN60/TWEEN60	DLS, SEM, TEM, AFM, ζ	Lipase	NR	NR	Enhancement of lipase-catalyzed hydrolysis in aqueous medium	[66]
NG-FACD nanoconjugates	Electrostatic assembly with folic acid-functionalized carbon dots	DLS, fluorescence and cellular assays	Riboflavin, paclitaxel	Enhanced loading relative to individual carriers	NR	Theranostic platform; targeted combination therapy and photodynamic effect	[69]
YYAYY covalent	Visible-light-induced covalent self-assembly mediated by ruthenium complex	TEM, high-resolution TEM	Gold or platinum precursors	NR	NR	Artificial nanoreactor; biomineralization and catalytic hybrid nanogels	[73]
Histidine-rich peptide NGs (H5, H9, H13)	Gamma-radiation	DLS, zeta potential	Fluorescent label	NR	NR	Nano-imaging platform for pancreatic cancer diagnosis	[74,78]

## 4. Conclusions

Peptide-based nanogels can represent and cover the role of emerging “hydrogel-to-nanoparticle” formulations. Unlike peptide hydrogels, whose application is limited to topical use, peptide nanogels, for their dimensions in the nanoscale range, can be easily injected in vivo, giving the opportunity to improve drug delivery by enabling precise control over pharmacokinetics, tissue exposure, and safety.

It is relevant to highlight that the term “*nanogel*” was initially used (and sometimes still used) to indicate nanostructured fibrillary peptide hydrogels [81,82,83]. Only successively, the terms were recalled to describe hydrogel nanoparticles. As reported by the examples discussed in this review, the peptide primary-sequence design, coupled with an appropriate formulation strategy, can drive both core structure and function, suggesting peptide NGs as especially attractive drug-delivery systems. Indeed, the chemical accessibility to peptide primary sequences allows a balancing of net charge, modulation of hydrophobicity and secondary-structure propensity, responsiveness against solvent, pH and ionic strength. The chemical features of peptides also induce a highly reproducible access to core–shell formulation with a dense fibrillary peptide core and a hydrophilic stabilizing corona, thus providing colloidal stability, reduced opsonization and modulation of the release profile. The architectural features described were also used for the delivery of both hydrophilic and hydrophobic active pharmaceutical ingredients. Compared with conventional polymeric NGs, peptide-based ones offer sequence-defined composition, intrinsic biodegradability into amino acids, high biocompatibility, and an easier introduction of bioactive and/or stimuli-responsive motifs. These modifications are often induced in milder self-assembly conditions with respect to polymeric NGs, reducing the need for potentially toxic synthetic cross-linkers or residual monomers. At the same time, however, the literature indicates that peptide-based NGs still lack clinical validation, compared to other nanoformulations, including NC-6004 (cisplatin micellar nanoparticle, phase I/II), NC-6300 (epirubicin micellar nanoparticle, phase I), and NK105 (paclitaxel micellar nanoparticle, phase III). This evidence underlines the limited clinical translation of gel-like nanoformulations and the most relevant requirements of manufacturing-controllable particle attributes in GMP protocols for an improvement of reproducibility and translation. According to these considerations, peptide NGs may be viewed as complementary, rather than simply alternative, to other nanoplatforms. For example, compared with liposomes, NGs generally provide a more hydrated and mechanically robust three-dimensional matrix, achieving slower and more tunable stimuli-triggered release, whereas liposomes retain the major advantage of decades of pharmaceutical development, standardized large-scale manufacturing, and multiple approved products, although they may suffer from membrane leakage, fusion, and formulation complexity for some cargos (e.g., genetic materials). Looking ahead, as for peptide hydrogel, [45] the main challenge for peptide-based NG formulations resides in their translation of their elegant supramolecular design into robust pharmaceutical products by improving batch-to-batch reproducibility, studying serum stability, storage stability, resuspension and freeze-drying processes, protein-corona effects, sterilization and scalable GMP-compliant manufacturing. An additional important limitation hampering the clinical translation of peptide-based nanogels is the lack of systematic data about their immunogenicity, since most available studies mainly address physicochemical characterization, cytotoxicity, cellular uptake, and only occasionally serum stability or hemocompatibility [50,51]. Additionally, the establishment of clear in vivo fate, pharmacokinetic profile, biodistribution, injection and safety, and regulatory benchmarks can move these systems from promising laboratory constructs to clinically credible nanomedicines.

## Data Availability

No new data were created or analyzed in this study. Data sharing is not applicable to this article.
